# First Description of the Extended Spectrum-Beta-Lactamase Gene *bla*_CTX-M-109_ in *Salmonella* Grumpensis Strains Isolated from Neonatal Nosocomial Infections in Dakar, Senegal

**DOI:** 10.1371/journal.pone.0157683

**Published:** 2016-06-29

**Authors:** Amadou Diop, Bissoume Sambe-Ba, Abdoulaye Seck, Mouhamadou Lamine Dia, Lassina Gadi Timbiné, Aïssatou Ameth Niang, El Hadji Momar Ndiaye, Mouhamadou Abdoulaye Sonko, Abdoul Aziz Wane, Raymond Bercion, Ousmane Ndiaye, Moussa Fafa Cissé, Amy Gassama-Sow

**Affiliations:** 1 Unité de Bactériologie Expérimentale (UBE), Institut Pasteur de Dakar, Dakar, Sénégal; 2 Laboratoire d’Analyses Biologiques et Médicales (LABM), Institut Pasteur de Dakar, Dakar, Sénégal; 3 Laboratoire de Bactériologie-Virologie Centre Hospitalier National de FANN, Dakar, Dakar, Sénégal; 4 Laboratoire de Bactériologie-Virologie Centre Hospitalier National d’Enfants Albert Royer de Fann Dakar, Dakar, Sénégal; 5 Unité de Néonatologie, Centre Hospitalier Abass Ndao, Dakar, Sénégal; 6 Faculté de Médecine, Pharmacie et d’Odontologie Université Cheikh Anta Diop de Dakar, Dakar, Sénégal; 7 Department de Génie Chimique et Biologie Appliquée, Ecole Supérieure Polytechnique, Université Cheikh Anta Diop de Dakar, Dakar, Sénégal; Aga Khan University Hospital Nairobi, KENYA

## Abstract

Nosocomial infections are very common in African hospitals, particularly in neonatal units. These infections are most often caused by bacteria such as *Escherichia coli*, *Klebsiella* spp and *Staphylococcus* spp. *Salmonella* strains are rarely involved in nosocomial infections. Here, we report the first description of *S*. Grumpensis in neonatal infections in Senegal. Seventeen *Salmonella* strains were isolated from hospitalized infants’ stool samples. The following resistance phenotype was described in strains: AMX^R^TIC^R^CF^R^ FOX^R^CFX^R^CTX^R^CAZ^R^IMP^S^ATM^R^NA^R^NOR^R^CIP^R^TM^R^GM^R^TE^R^SXT^R^. All isolates were susceptible to imipenem, 15 out of 17 produced an extended spectrum ß-lactamase (ESBL). *bla*_*OXA-1*_, *bla*_*SHV-1*_, *bla*_*TEM-1*_, *bla*_*CTX-M1*_ genes were detected in strains 8, 13, 5 and 8, respectively. *bla*_*CTX-M1*_ sequencing revealed the presence of *bla*CTX-M-109. Thirteen of the 17 *Salmonella* Grumpensis strains were analyzed by PFGE. These 13 isolates belonged to a single pulsotype and were genotypically identical. This is the first report of neonatal *S*. Grumpensis infections in Senegal, and the first report of *bla*CTX-M-109 in the genus *Salmonella*.

## Introduction

The emergence and spread of antimicrobial resistance is a major public health problem. Multidrug-resistant bacterial infections due to prolonged hospitalization, antibiotic treatment and poor hygiene are an increasingly prevalent cause of morbidity and mortality [1]. The frequency of hospital-acquired neonatal infections unrelated to non-typhoidal *Salmonella* is increasing. Therefore, hospital-acquired infections are both an important cause of mortality and a major economic burden [2].

*Salmonella* strains usually cause mild gastroenteritis but can also lead to serious infection, especially in vulnerable populations (young children, the elderly and immunocompromised individuals) [3].

Animals, both domestic and wild, are the main reservoir of non-typhoid *Salmonella* (NTS). Human NTS infections are frequently due to the consumption of contaminated cooked or raw meat or animal products (poultry, burgers, eggs) [4], or human-to-human transmission. Human contamination via the fecal-oral route, resulting from inadequate hygiene, is also not uncommon. NTSs are predominant among multidrug resistant (MDR) bacteria. They are a major cause of food-borne infections and often cause collective or individual nosocomial outbreaks in developing countries, in particular in pediatric settings [5; 6].

The main aim of this work was to characterize both the resistance and molecular characteristics of *Salmonella* Grumpensis strains isolated during a nosocomial infection outbreak in a neonatal unit of a University teaching Hospital Center in Dakar, Senegal, in 2011.

## Materials and Methods

Between March to May 2011, nosocomial infection occurred in the neonatal unit in the university teaching hospital (Abass Ndao Hospital in Dakar). The unit which has a capacity of 35–40 beds including a room with nine individual cribs as well as large cradles (each holding three or four babies) and an intensive care room for premature babies with five illuminated tables and an incubator. These tables can accommodate up to three babies. The medical staff in this unit during the study period consisted of one midwife, six assistant nurses, two nurses’ aides, five temporary support staff and ten temporary doctors. Such suboptimal working conditions may be the cause of neonatal infection. Stool samples were collected from 16 infants with acute gastroenteritis hospitalized in the neonatal unit of the Abass Ndao Hospital in Dakar together with one sample from a bottle of antiseptic (eosin) used in care of the newborn babies.

All samples were sent to Hôpital d’Enfants Albert Royer laboratory for testing.

### Ethics Statement

Consent could not obtained from the babies’ mothers as they were illiterate but a physician explained that the samples would be analyzed and, if a pathogen was identified, further studies would be carried out. Mothers also requested that all data would be analyzed anonymously and they all agreed in principle.

### Microbiological investigations

Environmental samples were tested at the Pasteur Institute Dakar (IPD): air samples from the reception room for newborns and hospital wards (pathology room, dining room and a room for preterm neonates delivered by caesarean section).

- Surface samples were taken onto agar in the various rooms of the neonatal unit and basins and door latches were swabbed.
- Water samples were taken from the various taps in the unit and unopened formula cans were sampled.
- Samples were taken of disinfectant solutions prepared on site.

Stool samples from 18 staff members were tested for *Salmonella* at IPD

### Bacterial isolates

Clinical isolates were obtained by standard procedures from *Salmonella Shigella* (SS) agar plates (Becton Dickinson, diagnostics). *Salmonella* strains were serotyped with polyvalent antisera using an agglutination test (Bio-Rad) at the National Center for *Enterobacteriaceae*, Pasteur Institute according to the White-Kauffmann-Le Minor method [7].

### Antimicrobial testing

Antibiotic susceptibility testing was performed by the disc diffusion method on Mueller Hinton agar according the standard recommendations of the French Society of Microbiology (CA-SFM, 2010) [8]. The following antibiotic discs were tested: ampicillin, amoxicillin+ acid clavulanic, ticarcillin, cefalotin, cefoxitin, cefuroxime, cefotaxim, imipenem, nalidixic acid, aztreonam, norfloxacin, ciprofloxacin, gentamycin, tetracycline, trimethoprim-sulfamethoxazole, streptomycin (Bio-Rad, France). For carbapenems, ertapenem (10 μg/ml) antibiotic discs were used (Bio-Rad, France). The zones of inhibition were measured to assess resistance or susceptibility.

### Molecular detection of integrons and resistance genes

Genomic DNA was extracted with the commercial Qiamp DNA Mini Kit (Qiagen, Courtaboeuf, France) according to the manufacturer’s recommendations. To detect the molecular determinants of resistance, PCR was carried out with specific primers for resistance genes including betalactamins, (*bla*_*OXA-1*_, *bla*_*SHV-1*_, *bla*_*TEM-1*_), cephalosporins (*CTX-M*_*1*_, *CTX-*M_2,_ *CTX-M*_*9*_) [9], quinolones (*gyrA*, *gyrB*, *parC*, *parE*); (*qnrA*, *qnrB*, *qnrS*) [10] and tetracycline (*tetA*, *tetB*, *tetC*, *tetG*) [11], and for integrons (*intI*1, *intI*2, *intI*3) [12]. *Salmonella* Havana 07–319 strain was used for PCR positive control for betalactams, *qnr* genes and class integrons.

### Sequencing

Sequencing was performed with the Big Dye terminator for the characterization of class1 integrons, and antibiotic resistance genes. DNA was sequenced in an automatic sequencer (ABI Prism 3100; Applied Genetic Biosystems) in both directions with the same PCR primers used for amplification of the target genes. Contig sequences were edited with Chromas Pro and compared in the BLAST program of the NCBI (http://www.ncbi.nlm.nih.gov/blast/BLAST).

### Molecular typing by pulsed field gel electrophoresis (PFGE)

Isolates were typed by pulsed field gel electrophoresis. In brief, DNA from *Salmonella* Grumpensis isolates was prepared in agarose plugs [13]. Bacterial cells were embedded in low-melting-point agarose (Biorad, Marnes-la-coquette) and lysed with lysis buffer containing lysozyme and proteinase K. DNA was digested at 37°C with 10U/μl of the restriction endonuclease *Xba I* (Biorad, Marnes-la-coquette, France). Digested DNA from *S*. Braenderup H9812 was loaded every five lanes as the molecular marker, as recommended by Pulse Net [14]. The digests were run at 6V/cmat at 14°C for 16 hours in a 1% agarose gel in 0.5X Tris-Borate-EDTA buffer using the Genepath system (Biorad, Marnes-la-coquette, France). The gel was stained with ethidium bromide and photographed on an ultraviolet trans-illuminator (GelDoc, Biorad, France). The restriction endonuclease digests were compared visually and isolates with the same pattern of bands (same number and molecular weight) were considered to be the same strain.

### Bacterial conjugation

Conjugation experiments were carried out with *E*. *coli* NalR as the recipient. The recipients were selected on Luria-Bertani (LB) medium containing streptomycin (25μg/ml), nalidixic acid (50μg/ml), trimethoprim (5μg/ml) and ampicillin (100μg/ml) and the donors were selected on LB containing only streptomycin and trimethoprim (BioMérieux, France). A total of 5 ml of fresh LB broth was inoculated with either recipient or donor bacteria and incubated for 24 h at 37°C. After overnight incubation, cultures were diluted at 1:50 (400μl in 20ml of LBB) and incubated at 37°C with strong agitation for 4 hours until they reached the logarithmic growth phase (OD 600nm = 0.6–0.8). Donor and recipient strains were then mixed at the following ratios 1:1; 1:2; 1:10 and incubated at 37°C for 3 hours. Transconjugants were selected on LB agar plates supplemented with streptomycin (25μg/ml), nalidixic acid (50μg/ml), trimethoprim (5μg/ml) and ampicillin (100μg/ml). PCR was performed on transconjugants to determine whether resistance genes had been transferred.

## Results

Multiresistant bacteria were detected in environmental samples: *Salmonella* Grumpensis in a sample taken from a bottle of antiseptic (eosin) used for the care of newborns, and other Gram negative bacteria, including *Salmonella* Brandenburg with a wild-type resistance phenotype in a sample from a sink in the nurses' locker room. Stool samples from the 18 caregivers were all negative for *Salmonella*.

All 17 isolates in this study were biochemically identified and serologically confirmed as *Salmonella enterica* serovar Grumpensis. Babies were empirically treated with syrup containing norfloxacin and metronidazole (10–20 mg/kg/day) or intravenous ampicillin and gentamicin (50–100 mg/kg/day). All the babies were cured.

### Antibiotic susceptibility

Ten (10) out of 17 (58.8%) isolates showed the following antibiotic resistance pattern AMX^R^TIC^R^CF^R^FOX^R^CFX^R^CTX^R^CAZ^R^IMP^S^ATM^R^NA^R^NOR^R^CIP^R^TM^R^GM^R^TE^R^SXT^R^, Fifteen isolates produced an extended spectrum ß-lactamase (ESBL). All isolates were susceptible to imipenem.

In conjugation experiments, recombinants acquired the same pattern of antibiotic resistance as strains of *Salmonella* Grumpensis. *intI1* and resistance genes were transferred intact to recombinant strains, suggesting that there was a block transfer of resistance through conjugative plasmids.

### Molecular typing

**Fig 1:**
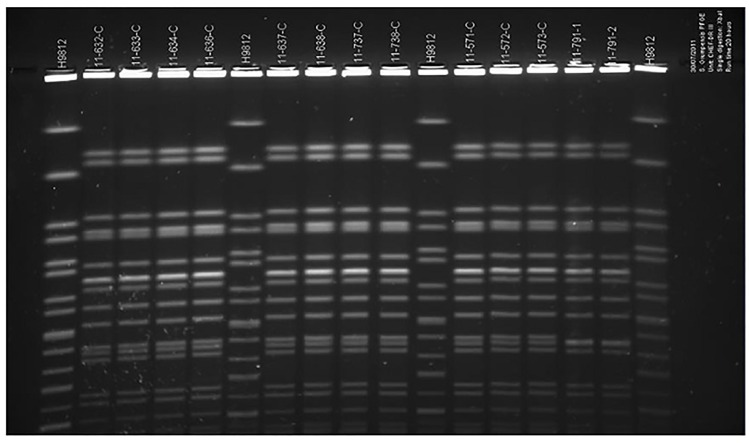
Representative PFGE patterns of *Salmonella* Grumpensis from infants with gastroenteritis.

Thirteen of the 17 strains of *Salmonella* Grumpensis were analyzed by PFGE. These 13 (12 from stool samples + 1from antiseptic sample) isolates belong to a single pulsotype and are genotypically identical (**Fig 1**).

### Detection of genetic determinants of resistance

All isolates were analyzed by PCR to search genetic determinants of resistance. Aminopenicillin resistance genes (*bla*_*OXA-1*_, *bla*_*SHV-1*_, *bla*_*TEM-1*_) were detected on 8, 13 and 5 strains, respectively, and all the 8 isolates (*bla*_*OXA-1*_) harbored genes encoding resistance to cephalosporins (*bla*CTX-M group 1). Sequencing has shown that 7/8 isolates with *bla*CTX-M group 1 carried the *CTX-M-109* gene. None of the strains harbored *bla*_CMY_ genes (the *ampC* gene). Quinolone resistance genes *gyrA* and *gyrB* were detected respectively in 8 (47%) and 14 (83%) isolates (Table 1). *parC* and *parE* genes were found respectively in one and 14 (82%) isolates, however no mutation was observed. *qnrB* and *qnrS* genes were found in 13 (76.5%) and 10 (59%) isolates, respectively (Table 1). All strains harbored the *qnrA* gene and were tetracycline resistant; however, the *tetA* gene was found in only 11 out of 17 (65%) strains. Class 1 integrons were present in 13 of the 17 strains (76.5%) and *aadA1* gene was carried (Table 1). Class 2 or class 3 integrons were not found.

**Table 1:** Phenotype and resistance genes.

Number of strains	Susceptibility patterns	Integrons	Resistance genes
8	AMX^R^ TIC^R^ CAZ^R^ CF^R^ FOX^R^ CTX^R^ CFX^R^		*bla*_*TEM-1*_
13	AMX^R^ TIC^R^ CAZ^R^ CF^R^ FOX^R^ CTX^R^ CFX^R^	*intI1*	*bla*_*SHV-1*,_ *aadA1*
5	AMX^R^ TIC^R^ CAZ^R^ CF^R^ FOX^R^ CTX^R^ CFX^R^		*blaOXA-1/CTX-M-109*
8	NA^R^ NOR^R^ CIP^R^		*gyrA*
14	NA^R^ NOR^R^ CIP^R^		*gyrB*
1	NA^R^ NOR^R^ CIP^R^		*parC*
14	NA^R^ NOR^R^ CIP^R^		*parE*
17	NA^R^ NOR^R^ CIP^R^		*qnrA*
13	NA^R^ NOR^R^ CIP^R^		*qnrB*
10	NA^R^ NOR^R^ CIP^R^		*qnrS*
11	TE^R^		*tetA*

^R^: resistance

## Discussion

During the study period, this neonatal nosocomial infection did not cause any deaths. No other case of nosocomial *Salmonella* Grumpensis infection was reported elsewhere.

Salmonellosis is rarely acquired in a hospital setting [15]. When it occurs, bacteria are usually transmitted by hand, care equipment and sometimes, antiseptic solutions [16]. Serotypes frequently involved in outbreaks in neonatology are *S*. Typhimurium, *S*. Mbao, *S*. Eimsbuettel, *S*. Heidelberg and *S*. Worthington [17]. S*almonella* outbreaks due to *S*. Ordain, *S*. Tel-el-Kebir, *S*. Mbao, *S*. Niloese *and S*. Poona have been reported in hospitals in Dakar [18]. This is the first report of neonatal *S*. Grumpensis infection in Senegal although this bacterium has been reported to cause infection in humans both in this country and elsewhere [19]. One *Salmonella* Grumpensis isolate was isolated from a bottle of antiseptic used for patient care reflecting poor hospital hygiene. Consistent with previous reports in the literature, we did not find *Salmonella* in the formula. Another source of *Salmonella* infection could be human-to-human transmission; despite extensive investigation, the source of the infection was never established.

The PFGE profiles of 13 isolates (including the strain from the eosin bottle) suggested that they all originated from the same clone.

The emergence of antimicrobial resistance within *Salmonella* species has been reported worldwide [20] and the proportion of resistant *Salmonella* strains is increasing in developing countries. *Salmonella* is naturally susceptible to the antibiotics routinely used to treat gastroenteritis. However, some strains produce ESBL, conferring resistance to aminopenicillins, cephalosporins, tetracycline and quinolones [21]. ESBL characterization revealed that eight isolates possessed *bla*_CTX-M-1_ genes, which are a common feature of *Enterobacteriaceae* [22]. *bla*CTX-M-1 PCR product sequencing showed that strains carried *CTX-M-109* gene. In Senegal, this is the first description of this gene in *Salmonella* even among *Enterobacteriaceae*. *CTX-M-109* gene was firstly described on *Shigella* species in Asia [23]. CTX-M producing *Enterobacteriaceae* strains are currently described in Senegal with *CTX-M-15* particularly common [24]. The *CTX-M-15* gene has been detected in Europe [25, 26], Asia [27], Africa [24, 28–29] and America [30]. CTX-M group 1 gene was detected in eight strains but the *bla*_CTXM9_ gene was not found in any of the strains. The absence of a *CMY* gene in our isolates suggests that the genetic determinants of resistance to beta-lactams were not located on the bacterial chromosome. Plasmids carrying the *bla*_*TEM*_ and *bla*_*SHV*_ genes, like those bearing the *bla*_*CTX-M*_ genes, often host resistance genes to other antibiotics (aminoglycosides, tetracycline, sulfonamides, trimethoprim and quinolone), and therefore may confer co-resistance similar to what we found in strains of *S*. Grumpensis. Furthermore, consistent with previous reports in the literature, some of our strains had group 1 *bla*_*CTX-M*_ genes and other resistance genes such as a betalactamase oxacillinase (*bla*_*OXA-1*_), which confers resistance to aminopenicillin [31]. Antibiotics are widely used in intensive care units, and this could explain the emergence of antimicrobial resistance in bacteria in the past ten years [32]. The resistance spectrum of *Salmonella* strains is determined by antibiotic use in animals, leading to selection pressure and the transfer of antibiotic resistance genes [33]. Strains that express CTX-M ß-lactamases are multidrug resistant. These strains have also plasmid-mediated genes conferring quinolone resistance [34]. The *gyrA* gene was detected in 8 strains and the *gyrB* gene in 14 strains. We found *qnrB* and *qnrS* genes among 13 and 10 isolates respectively but it was not determined whether they were *qnrB1*, *qnrB4*, or *qnrS1*.

Bacterial resistance to quinolones is typically mediated by changes in the target enzymes DNA gyrase (*gyrA* and *gyrB*) and topoisomerase IV (*parE* and *parC*) or by the modulation of drug entry and efflux. The role of plasmid genes (*qnrA*, *qnrB*, *qnrS*) encoding resistance to quinolones has been described in *Salmonella* [20]. The *qnr* gene originally emerged in *Enterobacteriaceae* [21]. The presence of plasmid genes (*qnrB*, *qnrS*) in 13 and 10 isolates explains the strong resistance of *Salmonella* to quinolones. The *qnr* genes are frequently described in *Salmonella* strains [35]. But in another study in Senegal, *qnr* genes were not detected. Only chromosomal genes such as *gyr* and *par* with mutations conferring resistance were described [36]. Overall, 65% of strains were resistant to tetracycline, probably because this antibiotic is so often used in animals [37]. However, the only tetracycline resistance gene found was the *tetA* gene; the *tetB* gene, which often confers resistance to tetracycline in *Enterobacteriaceae* was not found in isolates [38]. In our study, we identified only class 1 integrons among fourteen *Salmonella* isolates. Characterization of class 1 integrons revealed the presence of *aadA1* gene cassette encoding resistance to streptomycin and spectinomycin.

Conjugation experiments showed that all resistance genes were found in recombinants; therefore, "block" transfer of resistance genes occurred in the recipient strain of *E*. *coli*.

In addition, class 1 integrons from strains of *Salmonella* were transferred to *E*. *coli* during conjugation experiments. This suggests a plasmid origin for the genetic determinants of multidrug resistance among the strains of *S*. Grumpensis that caused outbreaks of gastroenteritis in the neonatal unit of Dakar Hospital. This means that multidrug resistance could rapidly spread and lead to treatment failure in many cases.

## Conclusion

This investigation detected strains of *Salmonella* Grumpensis that can cause nosocomial infections that could be particularly devastating in developing countries where the antibiotics used to treat them are expensive and difficult to come by.

*Salmonella* Grumpensis found in a bottle of antiseptic had a similar resistance profile to isolates from infected newborn babies. Pulsed-field gel electrophoresis of *Salmonella* Grumpensis strains found in the infants’ stools and the bottle of antiseptic confirmed the clonal link. Information about this outbreak in an intensive care unit is limited as the source of the contamination could not be identified; *mothers* and nurses were tested to investigate human-to-human transmission. The cleaning of surfaces in neonatal units is essential to remove multi-resistant germs, a simple way of controlling infection together with regular use of alcohol-based disinfectant solutions and training for care-givers. Establishment of an Infection Control service with adequate human and financial resources and the involvement of all personnel (staff awareness) are highly recommended.
